# NiCoCrAlX (X = Y, Hf and Si) Bond Coats by Cold Spray for High Temperature Applications

**DOI:** 10.1007/s11666-022-01322-2

**Published:** 2022-02-14

**Authors:** Cristian V. Cojocaru, Maniya Aghasibeig, Eric Irissou

**Affiliations:** National Research Council of Canada, Boucherville, QC Canada

**Keywords:** MCrAlY bond coats, cold spray processing, thermal barrier coatings (TBCs), high temperature applications

## Abstract

MCrAlX powder compositions (M = Ni,Co and X = Y, Hf, Si or combination) are often thermally sprayed via vacuum plasma spray (VPS), low pressure plasma spray (LPPS) or high velocity oxy-fuel to produce high temperature oxidation and hot corrosion resistant bond coats for thermal barrier coatings (TBCs). Cold spray technology is currently considered as a promising alternative to the traditional thermal spray solutions, having the advantage of delivering oxide-free and very dense metallic coatings at relatively lower costs compared to VPS and LPPS. NiCoCrAlY and NiCoCrAlYHfSi bond coats were deposited using a high pressure cold spray system and the influence of feedstock properties on the deposited bond coats were investigated. To improve NiCoCrAlYHfSi bond coat deposition, laser assisted cold spray (LACS) was employed. The results show that LACS can be successfully used to deposit this particular powder while eliminating nozzle erosion and low deposition efficiency disadvantages observed with conventional cold spray. To identify the optimal LACS setup for deposition of dense and uniform coatings, different laser/spray jet configurations were examined. TBCs with bond coats sprayed at the optimal configuration were assessed isothermally at 1150 °C in air for up to 500 h, and the results showed formation of a thermally grown oxide layer composed of predominantly Al_2_O_3_ with embedded small clusters of Hf-Y-rich oxides.

## Introduction

Thermally sprayed MCrAlY coatings are commonly used as bond coats in thermal barrier coatings to protect the underlying superalloy component of the gas turbine engine against high temperature oxidation and corrosion. When TBCs are exposed to high temperatures, a thin thermally grown oxide (TGO) layer forms at the bond coat and top coat interface due to oxygen diffusion through the ceramic top coat. This layer further protects the substrate by acting as a diffusion barrier and is composed of mainly α-alumina (Al_2_O_3_) and chromia (Cr_2_O_3_) as the preferred oxides and some other types of undesired and non-protective oxides, such as nickel oxide (NiO) and spinel type oxides ((Ni, Co)(Cr,Al)_2_O_4_) (Ref 1-5). Internal stresses within the TBC tend to increase by rapid and uneven growth of the TGO layer with the thermal exposure time, leading to cracking and spalling of the top coat. Therefore, growth of a stable, uniform, slow-growing and continuous TGO, mainly composed of Al_2_O_3_, is desired to improve TBC’s performance and durability (Ref 6, 7). Doping the MCrAlY bond coat material with reactive elements (RE) such as Hf, Re, Ta, Zr and Si in addition to yttrium is beneficial to the high temperature performance of the TBCs (Ref 3, 8-11). Addition of a RE dopant, in particular Hf, is reported to improve alumina scale adhesion and oxidation behavior (Ref 12, 13).

It has been shown that TGO characteristics such as composition and growth rate are highly influenced by the bond coat properties such as initial elemental composition, surface roughness, porosity and oxidation, which are highly dependent on the preparation process of the bond coat (Ref 5, 14, 15). The most widely used thermal spray processes for deposition of the MCrAlY bond coats include LPPS, VPS and HVOF, where the oxidation of the bond coat during manufacturing is reduced using inert gas conditions or lower jet flame temperatures (Ref 15-17). Although HVOF spraying of bond coats has gained much attention within the past several years due to the lower operating cost and superior coating oxidation behavior compared to VPS and LPPS, it has been demonstrated that surface oxides formed during the HVOF spraying process promote formation of the spinel type oxides, which are detrimental to the durability of the TBC (Ref 18-20).

Cold spray is a relatively recent spray technology that solves the oxidation problem associated with conventional thermal spraying processes. It involves acceleration of the injected powder particles (typically 1 to 50 µm) in a supersonic jet of compressed gas, typically nitrogen or helium, at temperatures lower than the melting point of the material. Upon high velocity impact on the targeted surface, the solid particles experience high strain rate deformation, building up an almost oxide-free and dense layer of the deposit material (Ref 21-24).

Cold spraying of MCrAlY bond coats was increasingly reported in the past years (Ref 25-27). Besides the presence of low oxide content within the bond coats sprayed by cold spray, crystallographic defects such as grain boundaries and dislocations induced by high velocity impact of the impinging particles facilitate Al diffusion in the coating and promote formation of a continuous and sustainable protective α-Al_2_O_3_ scale, leading to improved TBC durability and performance during high temperature oxidation (Ref 28, 29). In this respect, TBCs with cold spray bond coats exhibited superior oxidation behavior when compared to those with LPPS bond coats, where the latter showed formation of undesirable spinel type oxides (Ref 30).

MCrAlX bond coats are primarily cold sprayed with helium as the process gas as it can propel the particles to higher velocities due to its low density compared to nitrogen, resulting in higher deposition efficiencies (Ref 27, 30-34). However, helium is non-renewable and will drastically increase the process cost. Laser assisted cold spray (LACS) is another approach that can be used to improve the deposition of cold spray coatings. LACS is a hybrid deposition technique that combines cold spray process with material softening (powder and/or substrate) by a laser. The laser beam can be directed on the substrate directly under the nozzle (heating the substrate and the impacting powder simultaneously) or near the deposition area (heating the substrate). Increasing the temperature of the deposition zone using a laser allows particles to deform and deposit at lower impact velocities compared to the conventional cold spray, eliminating the need of using the expensive helium gas. Therefore, dense coatings can be made at high buildup rates and low operating costs (Ref 35-37). LACS is specifically beneficial for deposition of materials with high hardness and low sprayability (Ref 35, 38). Since the particles do not melt by the heat of the laser and deposit in solid state, oxidation remains comparable to cold spray.

In the present study, deposition of NiCoCrAlY bond coats was explored via high pressure cold spray technique using nitrogen as the propelling gas. The effect of addition of HfSi to the composition on deposition behavior of the cold sprayed bond coats was also investigated. LACS was used as a novel process to improve deposition of NiCoCrAlYHfSi bond coats. The effect of different laser-nozzle configurations on coating formation and the characteristics of their resulting macro and microstructures is discussed. TBC architectures were produced via deposition of APS-YSZ top coats onto these cold sprayed bond coats, and their high temperature oxidation performance was assessed through isothermal tests.

## Experimental

**Fig. 1:**
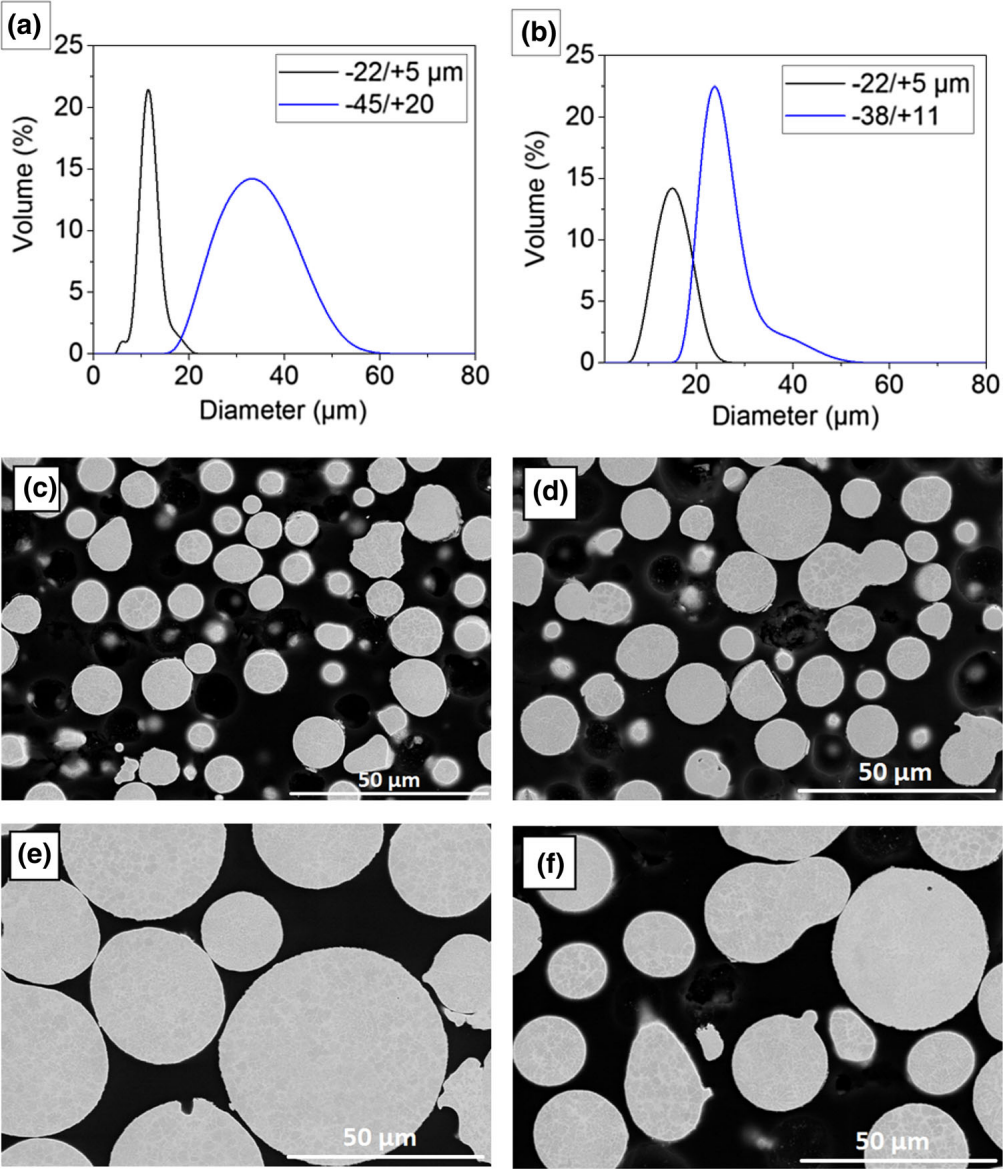
Particle size distribution and morphology of fine and coarse feedstock powders: (a, c, e) NiCoCrAlY and (b, d, f) NiCoCrAlYHfSi

Bond coats were cold sprayed using NiCoCrAlY powder (Amdry 365 type from Oerlikon Metco, USA) with two nominal particle size of − 45/+ 20 µm and − 22/+ 5 µm and NiCoCrAlYHfSi powder (Amdry 386 type from Oerlikon Metco, USA) with two nominal particle size of − 38/+ 11 µm and − 22/+ 5 µm. The chemical compositions of the powders, provided by the supplier are shown in Table 1. The particle size distribution of the powders was measured using a laser diffraction particle size analyzer (LS320, Beckman Coulter, USA). The morphology of the powders was characterized by using a scanning electron microscope (SEM) (S4700, Hitachi, Japan). The powders particle size distribution and morphology are shown in Fig. 1. The powders exhibit a spherical morphology characteristic to an atomized powder and cross-sectional micrographs clearly depict the 2 phase γ (matrix) / β (precipitates) characteristic to an MCrAlY alloy.

**Table 1:** Chemical analysis of the feedstock powders

Powder	Composition, wt.%						
	Co	Cr	Al	Ni	Y	Hf	Si
Amdry 365	23.45-23.55	17.04-17.13	12.06-12.45	Bal.	0.53-0.61	…	…
Amdry 386	21.79-22.47	16.76-17.34	12.28-12.69	Bal.	0.65-0.90	0.22-0.24	0.38-0.40

A high pressure cold spray (PCS-1000, Plasma Giken, Japan) system was used for deposition of the bond coats with both compositions onto grit-blasted Ø25.4 × 3.175 mm CMSX-4 superalloy disks, using the maximum propelling gas temperature and pressure of this system. Propelling gas temperature and pressure close to the operation limits of the system (i.e., 5 MPa and 1000 °C) give best coating density, adhesion and deposition efficiency for the MCrAlY-type alloy materials. However at these max conditions, with time, cermet nozzles that are usually used start to exhibit clogging due to material accumulation at the nozzle outlet. Therefore according to the recommendations of the equipment supplier, a glass nozzle is used as an alternative that alleviates the clogging yet in turn, in time, exhibiting erosion of the inner walls.

**Fig. 2:**
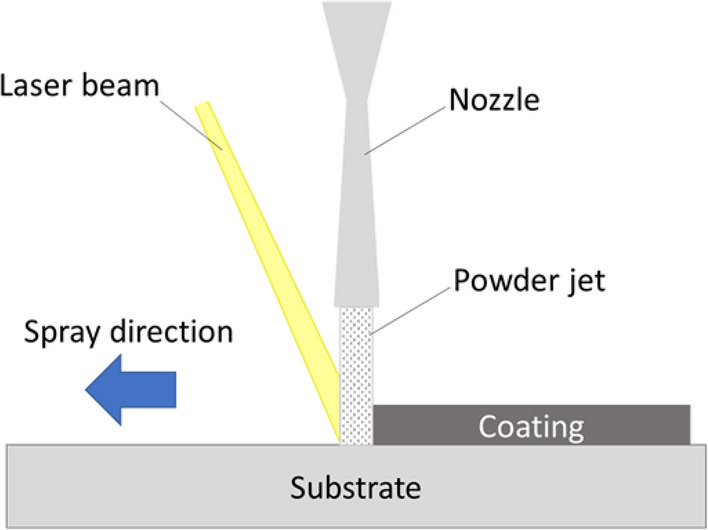
Schematic view of the LACS setup

NiCoCrAlYHfSi bond coats using the finer granulometry were also sprayed using a laser LACS setup composed of a 4 kW fiber laser (1064 nm wavelength, IPG Photonics, USA) coupled with a low pressure cold spray (Inovati_KM-CBS 2.2, USA) system. The laser was operated in the continuous wave mode and the laser spot and particle impact position from the nozzle were focused at the same spot on the surface of the substrate. Figure 2 shows a schematic view of the LACS setup. The effect of different nozzle-laser configurations by changing the angle in between the spray direction on the substrate and major axis of the incident laser beam on deposition properties of the bond coats were studied.

Atmospheric plasma spray (APS, 3MB, Oerlikon Metco, USA) was used for deposition of top coats to a thickness of ~400 µm onto top surface of the bond coats. ZrO_2_-8wt.%Y_2_O_3_ (YSZ) powder (Metco 204B-NS from Oerlikon Metco, USA) with nominal particle size of -75/+45 µm was used for deposition of the top coats. Spray conditions used for deposition of cold sprayed bond coats and APS top coats are shown in Table 2.

**Table 2:** Deposition parameters for bond and top coats

*Bond coat*	
High pressure cold spray	
Spray system	Plasma Giken PCS-1000
Propellant gas	Nitrogen
Gas temperature	1000 °C
Gas pressure	5 MPa
Standoff distance	25 mm
Gun traverse speed	200 mm/s
Step size	1 mm
LACS	
Spray system	Inovati_KM-CBS 2.2
Propellant gas	Nitrogen
Gas temperature	650 °C
Gas pressure	0.9 MPa
Standoff distance	25 mm
Gun traverse speed	50, 75 mm/s
Step size	0.25 mm
Laser system	4 kW fiber
Laser mode	Continuous wave
Power	500 W
Spot size	5.5 mm
*Top coat*	
APS	
Spray system	Oerlikon Metco, 3MB
Flow rate	Primary gas: nitrogen, 50 L/min Secondary gas: hydrogen, 10 L/min
Voltage	76 V
Current	500 A
Standoff distance	75 mm

APS (Mettech Axial III torch) and HVOF (Oerlikon Metco DJ2600-hybrid torch) were also employed to deposit bond coats using the coarse (− 45/+ 20µm) feedstock employing the spray conditions developed in a past research program (spray conditions are not presented here as they are proprietary and not the subject of this study). For a rapid and direct comparison of their performance all these TBC architectures were then isothermally oxidized at 1150 °C in air up to 500 h and 5 TBC replicas were tested for each architecture.

Coatings’ cross-sectional microstructures in as-sprayed condition and after isothermal tests were analyzed using SEM with an energy-dispersive x-ray spectrometer (EDX). XRD analysis using a Bruker D8 Discovery diffractometer (Bruker AXS, USA) with CuKα radiation was performed to evaluate the phase composition of the powders and as-sprayed bond coats.

## Results and Discussion

### Deposition of NiCoCrAlY Bond Coats

**Fig. 3:**
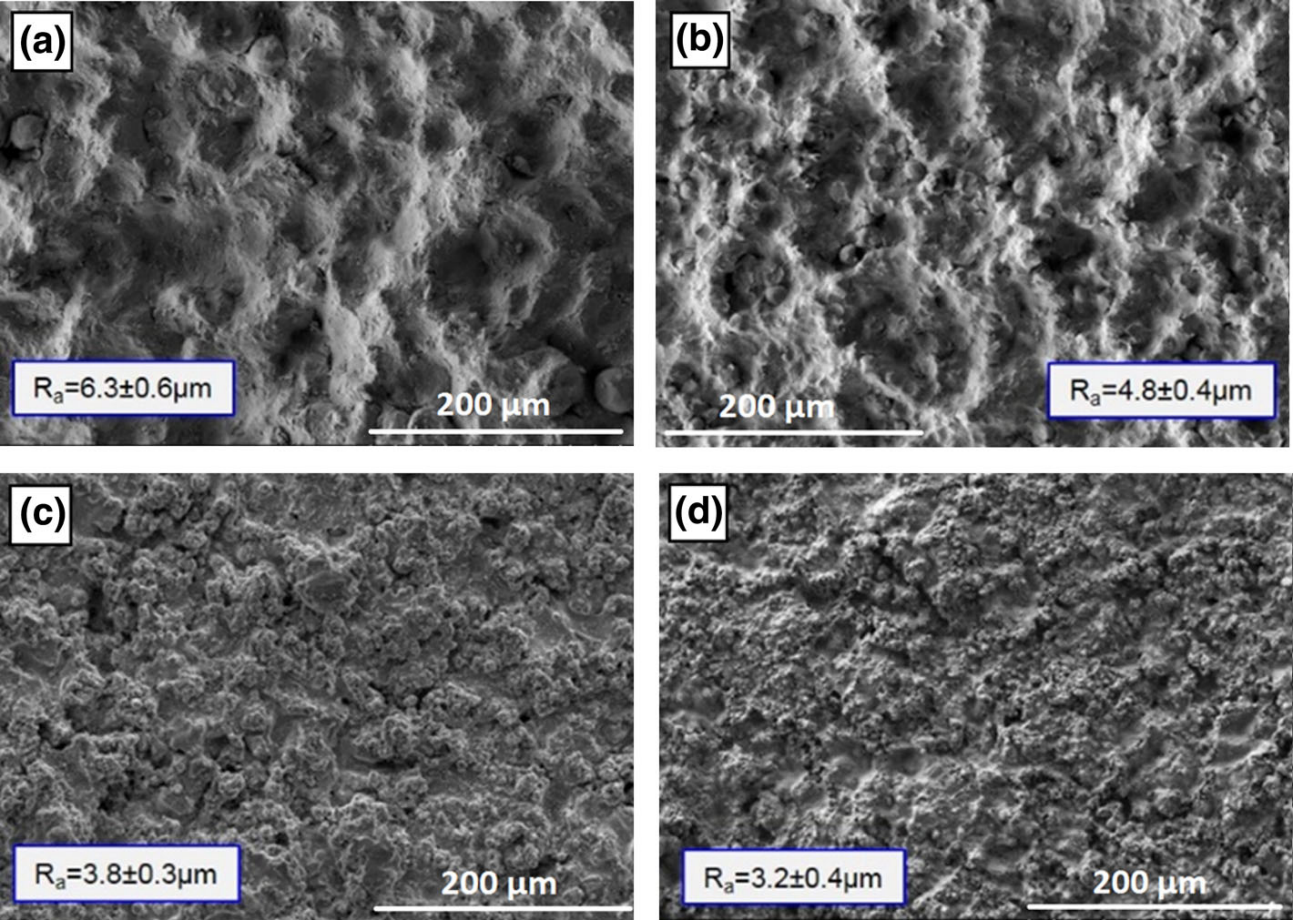
Surface morphology and roughness values of NiCoCrAlY coatings sprayed using (a) cold spray of coarse feedstock, (b) cold spray of fine feedstock (c) HVOF of coarse feedstock and (d) APS of coarse feedstock

**Fig. 4:**
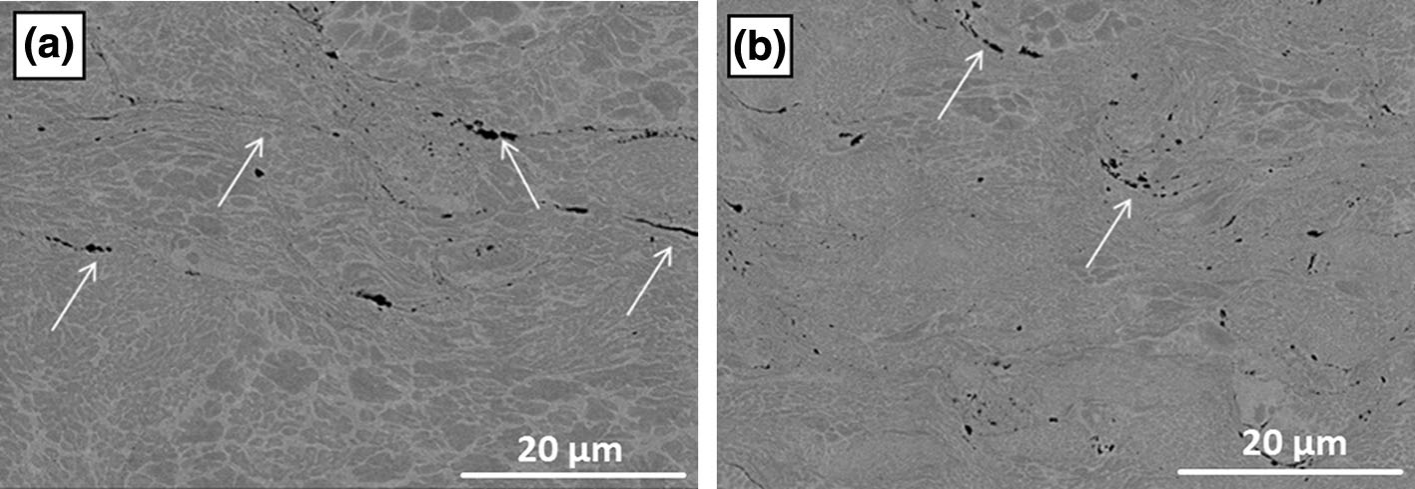
Cross-sectional microstructures of cold spray NiCoCrAlY coatings sprayed with (a) coarse and (b) fine feedstocks

Surface morphology of the NiCoCrAlY coatings sprayed by high pressure cold spray, HVOF and APS in the as-sprayed state and their respective surface roughness (Ra) are shown in the micrographs of Fig. 3. The presence of un-deformed particles and craters due to impact of particles that did not bind is visible on the top surface of the cold spray coatings. For both coarse and fine NiCoCrAlY feedstocks the roughness values of the cold spray deposited coatings were higher than the HVOF and APS coatings, prompting toward a better mechanical anchoring of the YSZ ceramic top coat. In addition, cold spray coatings sprayed using both coarse and fine powders appear well-consolidated while only few inter-particle vacancies and fine discontinuities at the interfaces were detected as shown by the arrows in the micrographs of Fig. 4. Higher velocity of the NiCoCrAlY fine particles at impact explains the slightly denser microstructure and presence of less particle interfaces for this coating (Fig. 4(b)). Particle velocity measurements taken with a Coldspray Meter (Tecnar, St-Bruno, QC, Canada) for standoff distances between 20 and 200 mm showed velocities in the range of 575 ± 25 m/s for the coarse powder cut and 725 ± 50 m/s for the fine size powder cut.

Diagnostic equipment currently available (e.g., Coldspray Meter from Tecnar or HiWatch from Oseir) can capture only the free in-flight powder velocities and can be used only in the absence of the substrate. However, both particle velocity and temperature at impact, as well as the substrate temperature are of paramount importance for determining proper coating deposition conditions. In the same time bow shock effect needs to be taken in consideration for fine particles that attempt to reach the substrate but are mostly deflected by the pressure bubble created under the gas jet stagnation point. Thus numerical simulation of the particle velocity and temperature at impact while taking into account the bow shock effect provides useful preliminary insights into the most appropriate spray conditions for reaching good deposits. The velocity and temperature of the NiCoCrAlY particles at impact were simulated using the computational fluid dynamics (CFD) module of the CSAM Digital Solutions software (National Research Council, Canada). This software allows accurate particle tracking from the nozzle inlet to the surface of the substrate by using complete flow profiles. SU2, an open source multiphysics and design software was used for the 2D axisymmetric compressible flow simulations. Having the particle diameter and volume fraction data from the particle size analysis, particle tracking simulations were then carried out using the one-way coupled particle computational fluid dynamics (CFD) module of the software. The influence of the substrate on the flow and bow shock effect on particle trajectory are considered in the simulations. The governing equations, their numerical discretization and the core elements of particle tracking are presented in (Ref 39). The simulation results showed that the average particle velocity and temperature were 585 m/s and 694 °C for the coarse feedstock and 763 m/s and 623 °C for the fine feedstock; revealing a close match with the velocity measurements recorded experimentally.

### Deposition of NiCoCrAlYHfSi Bond Coats

#### High Pressure Cold Spray

**Fig. 5:**
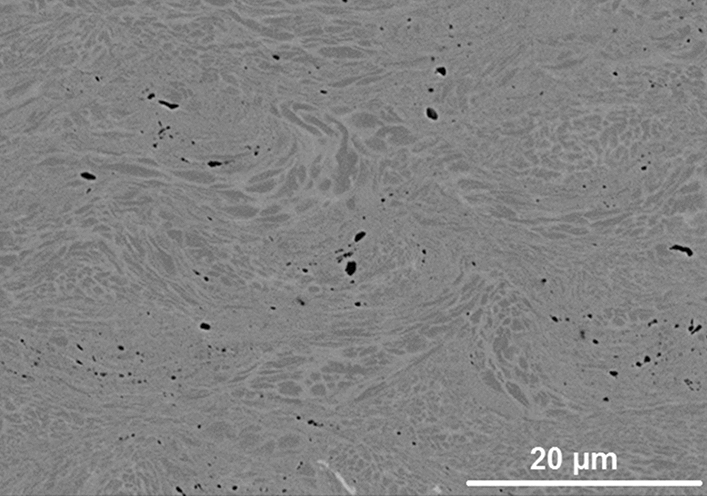
SEM micrograph of the as-sprayed NiCoCrAlYHfSi bond coat exhibiting the typical two-phase MCrAlY microstructure

Bond coats deposited using both granulometries of NiCoCrAlYHfSi showed very low deposition rates on CMSX-4 substrates demonstrated by low coatings thickness. In addition, extensive erosion and damage of the glass nozzle after just a few hours of spray was observed. This was mainly attributed to the hardness of the starting powders. Nano indentation measurements showed that NiCoCrAlYHfSi particles are about 23% harder than NiCoCrAlY powder particles (6.5 ± 0.3 GPa for NiCoCrAlYHfSi and 5.3 ± 0.5 GPa for NiCoCrAlY), thus showing lower cold sprayability and higher erosion behavior. In general only thin coatings of few tens of micrometers were obtained with the highest coating thickness of 50 µm reached when the coarse feedstock (− 38/+11 µm) was used. Figure 5 shows the SEM micrograph of such coating. The bond coat with less than 1% porosity is well-consolidated and shows no visible discontinuities such as adjacent particle interfaces. Similar to the NiCoCrAlY deposits, the as-sprayed microstructure appears to have retained the typical two-phase microstructure of γ matrix (light gray) and aluminum-rich β-NiAl precipitates (dark gray) that was initially observed in the feedstock.

In an attempt to improve the deposition, an admix of 50/50 wt.% of both granulometries of NiCoCrAlYHfSi and NiCoCrAlY powders were also sprayed. However, coating deposition remained similar to what was observed for the deposition of only NiCoCrAlYHfSi feedstock.

#### Laser Assisted Cold Spay (LACS)

Due to the limitation of depositing NiCoCrAlYHfSi bond coats with the required thickness of about 150 to 200 µm using the high pressure cold spray system, LACS was used as a novel solution to improve bond coats deposition efficiency. As described previously, LACS includes combining cold spray process with laser technology, where the heat input transferred by the laser on both substrate and powder and subsequent material softening allows deposition of thick and dense coatings at reduced impact velocities in a one-step process.

**Fig. 6:**
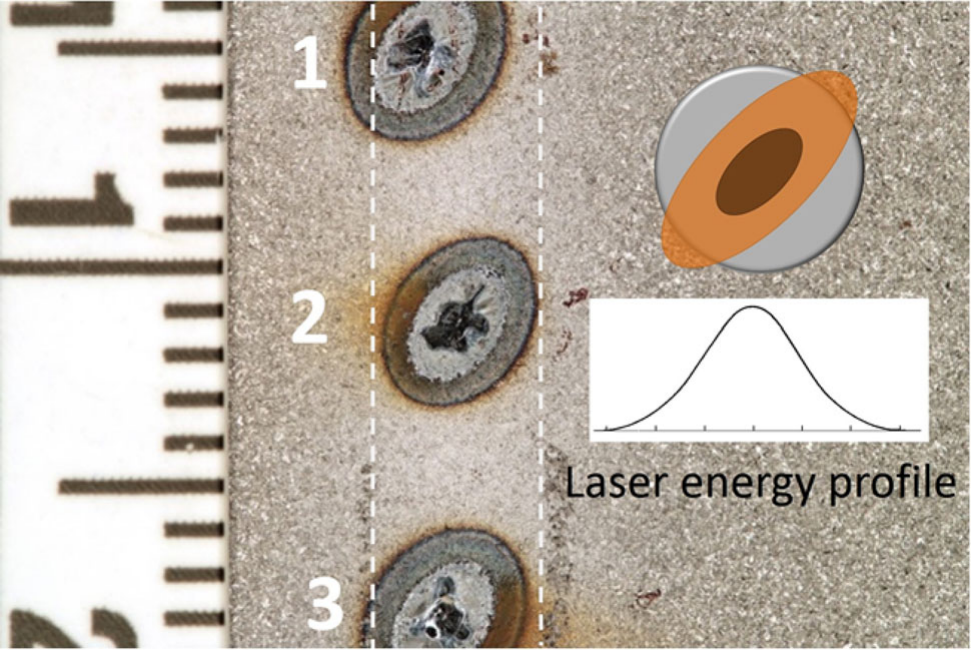
Alignment of the laser beam in regards to the gas jet

The setup was configured for the incident laser beam and the gas jet to meet at the same spot on the surface of the substrate (Fig. 2). To obtain the desired laser-nozzle alignment, the surface of the substrate was scanned by the gas jet leaving a discernible trace. A footprint of the laser beam was then marked on the trace by pulsing the laser on the surface. Figure 6 shows two malalignments (marked as 1 and 3) and one correct alignment (marked as 2) where the center of the elliptical footprint of the laser beam and the center of the gas jet trace overlap, thus the effect of the laser heat on the surface and the powder is maximized promoting powder deposition.

**Fig. 7:**
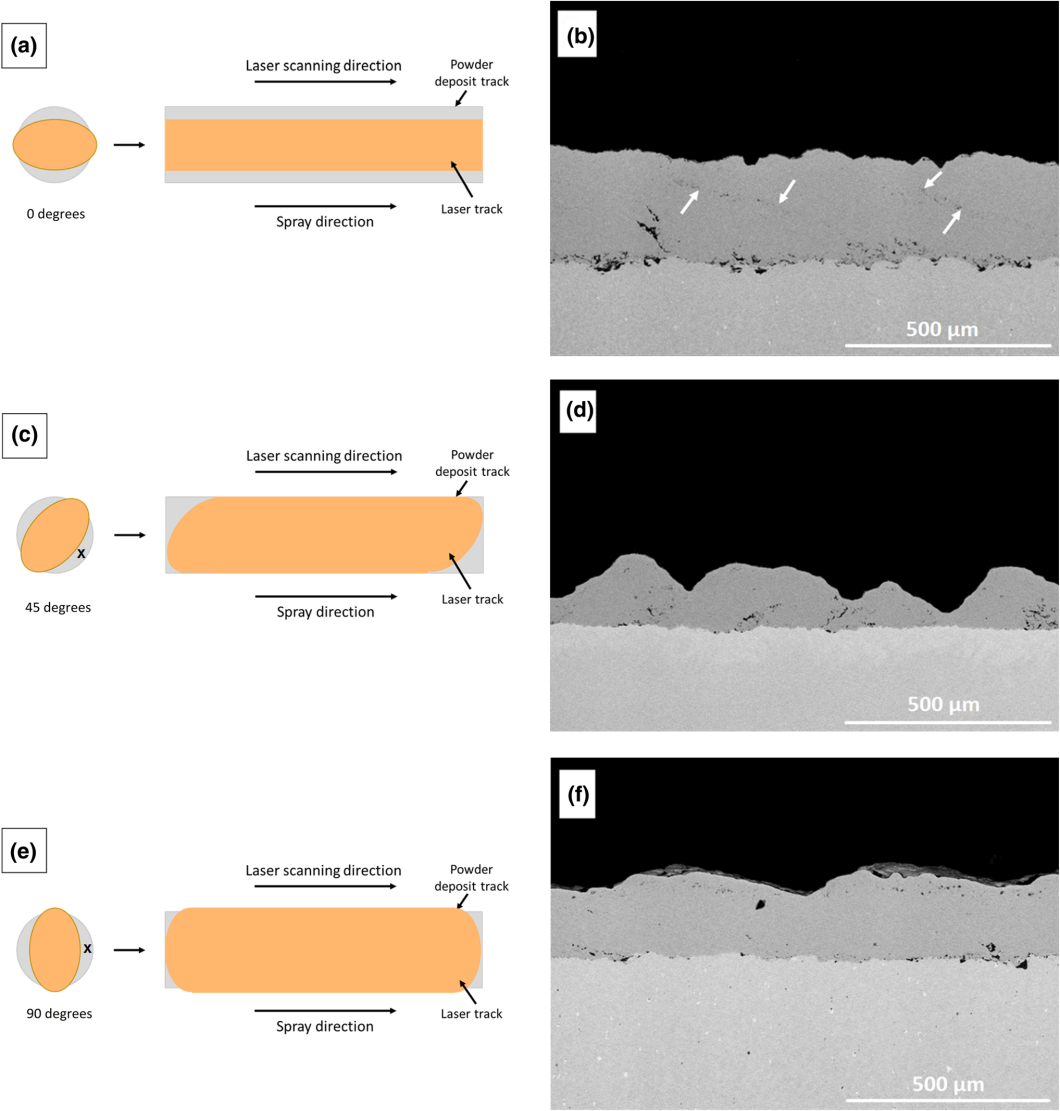
Different laser-nozzle configurations and the respective coatings obtained, where the angle in between the laser direction and spray direction were set at: (a, b) 0°, (c, d) 45° and (e, f) 90°

Three different nozzle-laser configurations were examined to find the best condition that results in the most continuous and uniform deposits. For these configurations, the angle between the major axis of the incident laser beam on the surface and the spray direction was set at 0, 45 and 90 degrees. The schematic view of each configuration and representative cross sections of the respective bond coats are shown in Fig. 7(a), (b), (c), (d), (e) and (f). It is noteworthy to emphasis on the fact that the cross sections were made perpendicular to the spray direction. It is apparent from the cross-sections that the bond coats deposited in all three configurations are well-consolidated containing limited levels of porosity (< 1%) or oxidation. Formation of dense microstructures can be attributed to high plastic deformation of the particles softened by the laser heat at high velocity impact on laser heated material (substrate or the previously deposited layers).

At 45 degrees configuration, the coating thickness is not uniform, whereas at 0 and 90 degrees configurations more uniform coatings were formed. Uniformity of the coating is influenced by the heating profile and overlapping of successive adjacent passes. As shown in Fig. 7(c), when the laser is oriented at 45 degrees with respect to the spray jet, there is an asymmetry in the deposition transverse to the spray direction. In this configuration, part of the jet particles impact the surface prior to the laser contact (region “x” in Fig. 7c). As it was presented in the previous section, these particles do not deposit or have minimal deposition on the surface. On the contrary, the particles that impact the surface at the same time or after the laser exposition will exhibit an enhanced deposition rate due to the heat induced by the laser. As region “x” is positioned toward the bottom section of the of spray jet on the surface, less material deposited in this region compared to the top section, resulting in the creation of thickness non-uniformity. As a result, when spraying subsequent passes, the particles deposit on an uneven surface, and thus the final overall thickness is non-uniform. Although region “x” is also present in the case of 90 degrees configuration, since the jet-laser configuration is symmetric, there is not clear thickness variation for this coating. For the 0 degree configuration, porosity was detected in between the adjacent passes as shown by white arrows in Fig. 7(b).

### Assessment of a TBC with LACS Bond Coat

**Fig. 8:**
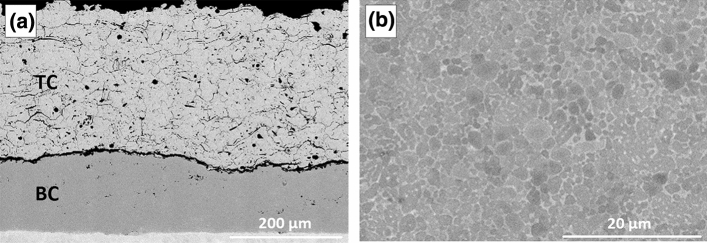
SEM microstructures of (a) as-sprayed TBC with LACS NiCoCrAlYHfSi bond coat and APS YSZ top coat and (b) LACS NiCoCrAlYHfSi bond coat

Figure 8(a) shows the microstructure of an as-sprayed TBC where the dense NiCoCrAlYHfSi bond coat was deposited using LACS with the configuration of 90 degrees between the incident beam and spray direction. The presence of a discontinuity at the interface between the bond coat and top coat indicates a weak adhesion mostly due to the low roughness of the bond coat surface (*R*_*a*_~ 2.5± 0.1 µm). As the TBC was intact after the spray of the YSZ top coat, it is presumed that the delamination was occurred during the metallographic preparation process. Lower surface roughness of this coating deposited by LACS compared to the coatings deposited by the high pressure cold spray system can be explained by particle softening and more severe deformation during deposition due to the heat induced by the laser.

However, it can be observed from the high magnification image of the bond coat of Fig. 8(b) that the microstructure was not affected by heating from the laser and remains similar to the microstructure of the starting powder with the presence of both β and γ phases. EDX analysis for identification of these two phases is shown in Table 3.

**Table 3:** EDX analysis of β phase and γ matrix within the LACS deposited NiCoCrAlYHfSi bond coat

Phase	Composition, wt.%					
	Co	Cr	Al	Ni	Hf	Si
β-phase	19.8	12.9	17.4	Bal.	0.2	0.4
γ-phase	24.3	22.1	8.7	Bal.	0.8	0.8

**Fig. 9:**
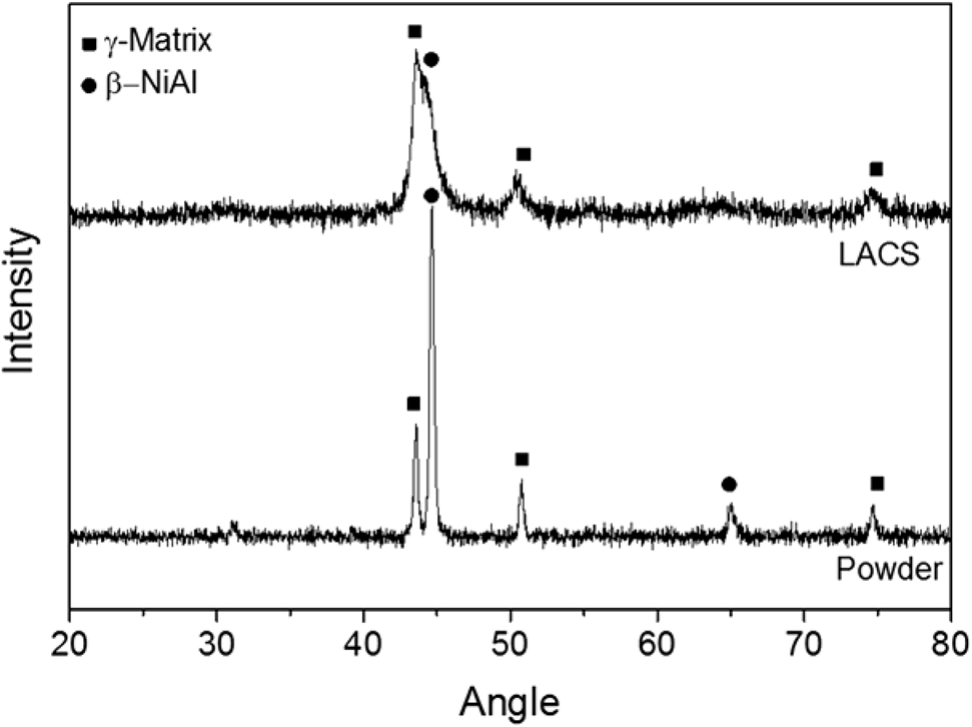
XRD spectra of the feedstock powder and LACS deposited bond coat demonstrating the two-phase microstructure

**Fig. 10:**
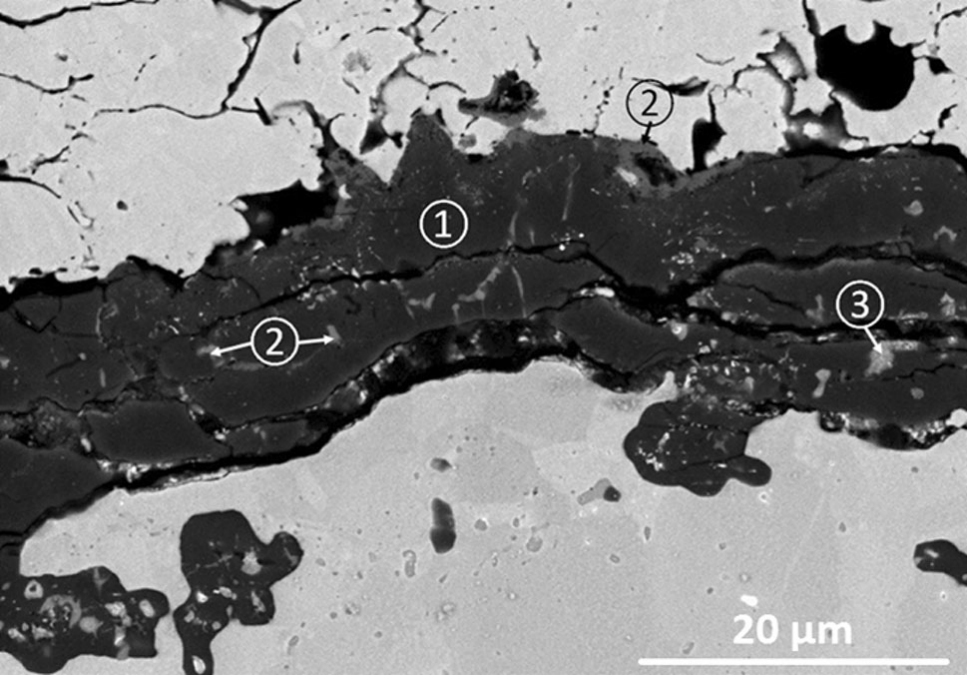
SEM micrograph of the TGO formed after 300 h of isothermal exposure at 1150 °C in air

The XRD analysis carried out on the feedstock powder and top surface of the LACS deposited bond coat is presented in Fig. 9. The pattern of the powder further demonstrates the presence of β and γ phases. For the LACS deposited bond coat, oxidation did not occur by the heat of laser as the particles did not melt but deposited in solid state. For the powder, β was identified as the dominant phase, whereas in the bond coat γ was identified as the dominant phase. Loss of β phase in the cold sprayed coatings was previously observed by other researchers and it was related to the dissolution of β-precipitates into the γ-matrix resulting from severe plastic deformation of the particles upon impact (Ref 5, 40). For the LACS coating, dissolution was not complete and partial dissolution of the β phase has occurred as shown by the XRD analysis and SEM image in Fig. 8(b). The LACS based TBC was then subjected to isothermal heat treatment at 1150 °C in air. After 300 h, spallation of the YSZ ceramic top coat was observed and failure occurred (more than 25% of the coating spalled off). Figure 10 shows the TGO layer that was detected at the interface of the bond and top coats after the isothermal exposure. EDX analysis revealed that the TGO is composed of a layer of predominantly Al_2_O_3_ as depicted by the dark gray phase marked as region 1. Mix oxides could be detected as a thin layer on the top surface of the TGO as well as small regions within the alumina scale (region 2). Some small regions of Hf-Y-rich oxides could also be detected within the TGO (region 3). Since LACS promoted the growth of mainly alumina-based TGO, failure of this TBC after 300 h was related to the low adhesion in between the top and the bond coat as it is suggested by the presence of the discontinuity in between these two layers in the as-sprayed microstructure (Fig. 8a) due to the low surface roughness of the as-sprayed bond coat.

## Conclusions

NiCoCrAlY feedstocks with two different granulometries were successfully deposited using a high pressure cold spray system. In both cases, the coatings appeared to be very well-consolidated while only few inter-particle vacancies were detected. In general, the surface roughness of the coatings deposited by cold spray were higher than those obtained by using either APS or HVOF when the same feedstocks were employed. Isothermal testing (1150 °C, in air) of the TBCs with cold sprayed bond coats showed endurance of more than 500 h without failure.

Addition of the HfSi to the feedstock composition resulted in deposition of coatings with minimal thickness and led to the erosion of the glass nozzle employed. To alleviate the challenge for depositing this powder, LACS based on combination of a laser and a low pressure cold spray system was employed. Different laser/ spray jet configurations were examined to determine the optimal strategy for deposition of a uniform coating. The coating sprayed at the 90 degree configuration exhibited uniform thickness and high density. Although the TGO composition shows predominantly Al_2_O_3_, the low roughness of this coating imposed by severe plastic deformation of the particles softened by the laser heat, did not promote an extended lifetime of the TBC. A strategy for increasing the surface roughness of the LACS bond coats needs to be developed, so that these TBCs would reach a lifetime comparable to those produced with conventional high pressure cold spray.
